# Myeloid dendritic cells are increased in the lesional skin and associated with pruritus in patients with prurigo nodularis

**DOI:** 10.1002/mco2.204

**Published:** 2023-02-11

**Authors:** Taoming Liu, Yuqi Chu, Sheng Li, Yuqian Wang, Xinyue Zhong, Hong Fang, Jianjun Qiao

**Affiliations:** ^1^ Department of Dermatology, The First Affiliated Hospital Zhejiang University School of Medicine Hangzhou China

## Abstract

The number of dermal mDCs (CD11c^+^ cells) was increased with itch intensity (*r* = 0.886, *p* = 0.003) using immunofluorescence (IF). On IF, CD11c^+^ mDCs expressed IL‐31 in lesional PN skin. Fluorescence in situ hybridization combined with IF also confirmed IL‐31 mRNA expression by mDCs in PN lesion. Higher population of colocalization of CD11c^+^ mDCs expressing IL‐31 mRNA were more than CD68^+^ macrophages and CD3^+^ T cells in consecutive sections of PN skin lesion. HC, healthy control; PN, prurigo nodularis; SPN, PN with severe pruritus (itch NRS score ≥7 points); MPN, PN with mild pruritus (itch NRS score <3 points); NRS, Numeric Rating Scale; AD, atopic dermatitis; mDC, myeloid dendritic cell.

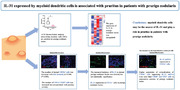

Dear Editor,

Prurigo nodularis (PN) is a chronic debilitating inflammatory skin disease, characterized by itch nodules on the trunk and extremities. The pruritus intensity tends to be higher in patients with PN than in patients with chronic skin diseases, including pruritic psoriasis, chronic urticaria, and atopic dermatitis (AD). Previous histologic reports indicated that PN lesion was characterized by abnormal sensory nerve fibers and cells, including keratinocytes, eosinophils, T cells, and mast cells.^1^, ^2^ The data demonstrated that various genes in PN lesion decreased to normal level after nemolizumab (anti‐IL‐31RA antibody) treatment, including *IL22, STAT1, STAT3, IL1B, IL1A, IL36G, DEFB4*, and *IL36A*.^1^ It was suggested that PN had a predominant Th22/IL‐22 profile.^2^ However, the mechanism of chronic itch in patients with PN remains unclear.

IL‐31 is a pro‐inflammatory cytokine of the IL‐6‐derived cytokine family, whose levels are highly correlated with itch severity. It signals through a heterodimeric receptor, consisting of IL‐31 receptor A (IL‐31RA) and oncostatin M receptor (OSMR), linking immune cells to the neuronal network. IL‐31 is expressed on a variety of immune cells in inflammatory skin lesions, including T cells, mast cells, eosinophils, and macrophages. In addition, the colocalization of IL‐4 and IL‐31 in CD11c^+^ myeloid dendritic cells (mDCs) has also been observed in dermatomyositis lesions.^3^ It is currently unclear whether dendritic cells (DCs) play a role in PN.

In the current research, we sought to investigate major inflammatory cells and itch mediators in PN lesions. We further quantified the immune cell populations and its relationship with itch severity, and also evaluated the cellular source of key itch mediators in PN lesions.

For studies using human participants, written informed consent was obtained from participants to participate in the study. This study protocol was reviewed and approved by the institutional ethics committees of the First Affiliated Hospital, Zhejiang University School of Medicine (approval number IIT‐2022‐571).

To identify the major immune cells infiltrating the PN skin, we estimated our transcriptomic data (lesional and perilesional PN skin) using the xCell scoring online tool. RNA‐seq analysis was conducted on a panel of 17 patients and six healthy control (HC) skin samples, including lesional and perilesional PN skin (*n* = 7), lesional AD skin (*n* = 5), lesional psoriasis skin (*n* = 5), and HC normal skin (*n* = 6). For further demographic information, see Tables S1 and S2. xCell analysis of lesional and perilesional PN skin marked differences in DCs compartment, indicating DCs appear to be predominant in PN (Figure 1A and Figure S1). However, within DCs, there were no marked changes in plasmacytoid DCs subpopulations (Wilcoxon *p* = 0.063, Figure S1B).

**FIGURE 1:**
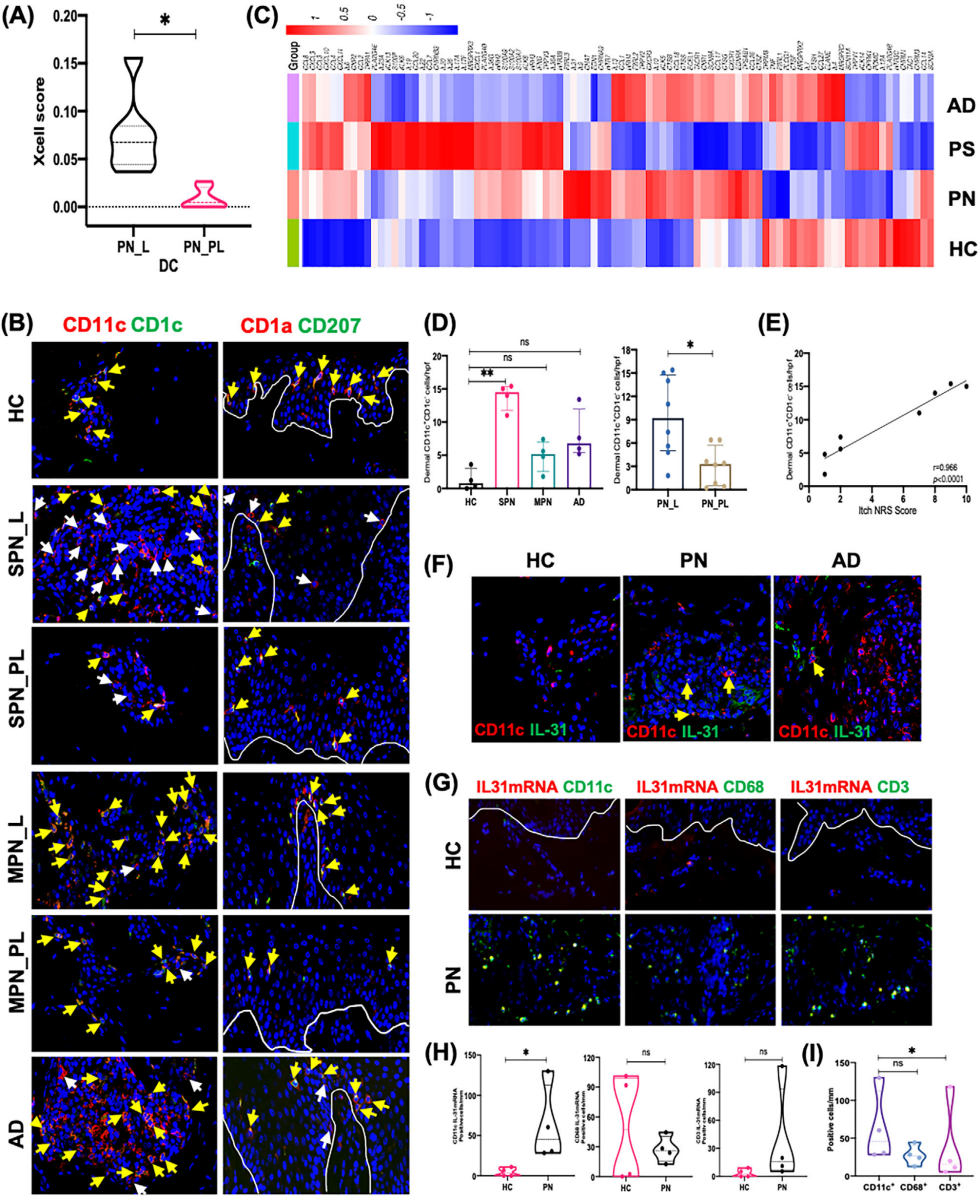
(A) xCell analysis demonstrated changes in dendritic cell gene signatures in lesional prurigo nodularis (PN_L) and perilesional PN (PN_PL) skin. *p < 0.05 by Wilcoxon matched‐paired test. (B) Representative CD11c (red), CD1c (green) and CD1a (red), CD207 (green) immunofluorescence in healthy control (HC), PN with severe pruritus (SPN; itch Numeric Rating Scale [NRS] score≥7 points), PN with mild pruritus (MPN; itch NRS score <3 points), and atopic dermatitis (AD) human specimens, respectively. The white arrows indicate resident myeloid dendritic cells (mDCs) or inflammatory dendritic epidermal cells (IDECs). The yellow arrows indicate inflammatory mDCs or Langerhans cells (LCs). Dotted lines mark the basement membrane separating the epidermis from the dermis. (C) Heatmap of itch‐associated genes in HC (*n* = 6), PN (*n* = 7), psoriasis (PS; *n* = 5), and AD (*n* = 5) samples revealed by transcriptomic RNA‐seq data analysis. (D) Quantitation of CD11c^+^CD1c^−^ inflammatory mDCs in each high power field (HPF) image of the dermis (*n* = 4, per group). *n* = 4, per group, **p < 0.001 by Kruskal‒Wallis test; *n* = 8, *p < 0.05 by Wilcoxon matched‐paired test; ns, not significant. (E) Correlation between itch NRS score and the number of inflammatory mDCs. (F) Representative CD11c (red) and IL‐31 (green) immunofluorescence in the dermis of HC, PN, and AD skin samples. The yellow arrows indicate IL‐31 expression in mDCs in the dermis. (G) Representative immunofluorescence CD11c (green), CD68 (green), and CD3 (green) and fluorescence in situ hybridization of IL‐31mRNA (red) in the dermal skin from HC and PN samples. (H) Quantification of CD11c^+^IL‐31^+^ cells, CD68^+^IL‐31^+^ cells, and CD3^+^IL‐31^+^ cells in HC (*n* = 4) and PN (n = 4) samples. (I) Quantification of CD11c^+^IL‐31^+^ cells, CD68^+^IL‐31^+^ cells, and CD3^+^IL‐31^+^ cells in PN (*n* = 4) samples. The levels were analyzed using Image J and averaged across five randomly selected HPF images per skin sample. All data are expressed as the median with interquartile range. Nuclei were stained with DAPI (blue).

Under the inflammatory state, there are two main subtypes of mDCs in the dermis, including resident mDCs (CD11c^+^CD1c^+^ cells) and inflammatory mDCs (CD11c^+^CD1c^−^ cells). We found significant dermal CD11c^+^ mDC infiltration in the PN lesions, particularly inflammatory mDCs (Figure 1B and Figure S2). In the dermis, numbers of inflammatory mDCs were increased and significantly correlated with pruritus intensity (*r* = 0.966, *p* < 0.0001; Figure 1D,E). Therefore, we further hypothesized that mDCs are important in PN. Though mDCs have not been reported previously in PN, several studies on similar infiltrating cells in inflammatory skin diseases, including psoriasis, cutaneous lupus erythematosus, and dermatomyositis, have been performed. These DCs may have important actions in antigen presentation and bridging the immune systems and peripheral sensory neurons.

To further characterize epidermal DCs, we investigated the two possible subtypes of DCs in lesional PN skin. In addition to resident Langerhans cells (LCs), inflammatory dendritic epidermal cells (IDECs) were also present in the epidermis in the inflammatory state. Epidermal CD1a^+^CD207^−^ cells were observed both in lesional PN and AD skin (Figure 1B). Similar numbers of epidermal LCs were detected in lesional PN, lesional AD, and HC normal skin (Figure S3A). IDECs were increased both in lesional PN and AD skin compared with HC normal skin (*p* = 0.047 and 0.022; Figure S3A). However, the number of IDECs in lesional PN skin did not correlate with itch numeric rating scale score of patients with PN (*r* = 0.560, *p* = 0.150; Figure S3B). A previous study showed that the numbers of epidermal HLA‐DR^+^ and S‐100^+^ cells (LCs) in PN lesions were decreased in five but increased in two cases.^4^ The discrepancy in the reported number of LCs may result from the use of different staining markers, experimental protocols, and quantification methods to evaluate LCs by immunohistochemical and immunofluorescence (IF) staining. Additionally, it is needed to investigate whether clinical heterogeneity affects the number of LCs in PN lesional skin, including different parts, degree of inflammatory infiltration, and degree of aging of skin samples. A larger sample size is necessary. We found that the number of IDECs was increased in lesional PN skin. However, the number of IDECs did not correlate with itch intensity. The role of IDECs in PN remains elusive; therefore, future studies will aim to elucidate this.

We identified the RNA‐seq profile for chronic pruritus in PN, AD, psoriasis, and HC by comparing differentially expressed genes (Figure 1C). Our data suggested that the gene transcripts of IL‐31 were elevated in lesional PN skin. Compared to HC, AD, and psoriasis, only a few pruritus‐related genes were overexpressed in lesional PN skin, including *F2RL3*, *IL31*, *IL9*, *CHAT*, *EDN1* (endothelin‐1), *CHRNA9*, and *HTR7*. We further examined the IL‐31 expression in patients with PN. However, there were no marked differences in both epidermal and dermal IL‐31 expressions in lesional PN skin compared with lesional AD, lesional psoriasis, and normal skin from HC (Figure S4A,B), consistent with previous reports.^5^ Serum levels of proinflammatory cytokines were not significantly different between HC and patients with PN, including IL‐6, IL‐10, IL‐2, IL‐31, IL‐5, IL‐13, IL‐17A, IL‐17F, IL‐22, and IFN‐γ (Figure S5). TNF‐α and IL‐4 serum levels were lower in patients with PN than in HC.

We then became interested to address the source of IL‐31 in lesional PN dermis. Given that the number of dermal mDCs increased with itch severity, we further hypothesized that mDCs may additionally be a key producer of IL‐31. We observed through IF staining that IL‐31 was expressed by CD11c^+^ mDCs in PN lesions (Figure 1F). In combined fluorescence in situ hybridization (FISH) and IF analyses, IL‐31 mRNA colocalized with CD11c^+^ mDCs in PN lesions at the gene level (Figure 1G,H). Most IL‐31 mRNA expression appeared to be in the dermis in PN. However, IF staining showed IL‐31 expression both in the epidermis and dermis in PN. This different result may be because of differences in the technique of detecting mRNA versus protein. Additional study will be needed to confirm these findings regarding the location and numbers of cytokine production.

To explore the major producer of IL‐31 in PN lesions, we next quantified IL‐31 mRNA expressed in CD11c^+^ mDCs, CD68^+^ macrophages, and CD3^+^ T cells in consecutive PN lesion sections. Combined FISH and IF analysis illustrated increased IL‐31 mRNA expression from CD11c^+^ mDCs compared with that from CD3^+^ T cells (*p* = 0.04; Figure 1I). These results suggest that mDCs were the likely source of IL‐31 and may be important in PN development.

There are a few limitations to this study. First, our sample size was relatively small. Second, although we found that mDCs may be a major source of IL‐31, we did not survey this through a dynamic assay, and this will be our future research focus.

In conclusion, we demonstrated that increased numbers of mDCs in PN lesions related to itch intensity in patients with PN. mDCs may be a major source of IL‐31 and appear to play a role in the development of pruritus in PN.

## AUTHOR CONTRIBUTIONS

Taoming Liu and Jianjun Qiao contributed to designing the study, investigation, and manuscript writing. Yuqi Chu contributed to investigation. Sheng Li contributed to resources. Yuqian Wang contributed to visualization. Xinyue Zhong contributed to data curation. Jianjun Qiao and Hong Fang contributed to project administration and funding acquisition. Taoming Liu and Jianjun Qiao manuscript revision and read the submitted reversion. All authors have reviewed and approved the final version of the manuscript.

## CONFLICT OF INTEREST

The authors declare they have no conflicts of interest.

## FUNDING STATEMENT

This work was supported by National Natural Science Foundation of China (82173400, 81972931) and Medical and Health Science and Technology Project of Health Commission of Zhejiang Province (2020KY558).

## ETHICS STATEMENT

This study protocol was reviewed and approved by the institutional ethics committees of the First Affiliated Hospital, Zhejiang University School of Medicine (approval number IIT‐2022‐571).

## Supporting information



Supporting Information

## Data Availability

All data generated or analyzed during this study are included in this article. Further enquiries can be directed to the corresponding author.
