# Supplementary material for: Myeloid dendritic cells are increased in the lesional skin and associated with pruritus in patients with prurigo nodularis
Source: MedComm (2020). 2023 Feb 11;4(1):e204. doi: 10.1002/mco2.204 (PMC9921813; doi:10.1002/mco2.204)
Supplement: Supplementary file 1 — Supporting Information [file MCO2-4-e204-s001.docx]

Supplementary Materials for

Myeloid dendritic cells are increased in the lesional skin and associated with pruritus in patients with prurigo nodularis

Taoming Liu^#^, Yuqi Chu^#^, Sheng Li, Yuqian Wang, Xinyue Zhong, Hong Fang^*^, Jianjun Qiao^*^

Correspondence to: Jianjun Qiao: qiaojianjun@zju.edu.cn; Hong Fang: fanghongzy@zju.edu.cn;

**This PDF file includes:**

Materials and Methods

References

Figure S1 to S5

Table S1 to S2

**MATERIALS AND METHODS**

**Clinical samples**

The study was approved by the Ethics Committee of the First Affiliated Hospital, Zhejiang University School of Medicine (Approved number: IIT-2022-571). This study was performed according to the Declaration of Helsinki. All volunteers and patients provided written informed consent.

*Human skin specimens*

Full-thickness skin biopsies (3 mm thickness) were obtained from heathy volunteers, lesions and peri-lesions of patients with prurigo nodularis (PN), and lesions of patients with atopic dermatitis (AD) and psoriasis (Table S1). Healthy volunteers had no atopic background. According to the criteria of Hanifin and Rajka, patients with AD were included in the study.[^1^](#_ENREF_1) The diagnosis of PN was made by two experienced dermatologists according to the criteria of Shawn.[^2^](#_ENREF_2) The degree of pruritus was assessed by numeric rating scale (NRS), recording the pruritus peak within 24 h before consultation; 0 represents no pruritus, and 10 refers to the worst pruritus. According to itch intensity, patients with PN were divided into three groups: severe itch (itch NRS score ≥7 points), moderate itch (itch NRS score 3-6 points), and mild itch (itch NRS score 1-2 points).[^3^](#_ENREF_3) All the skin samples were obtained from UV-non-exposed body sites in the First Affiliated Hospital, Zhejiang University School of Medicine.

*Human serum samples*

Human serum was acquired from the First Affiliated Hospital, Zhejiang University School of Medicine. Peripheral blood samples were collected from patients with PN (n=24), patients with AD (n=13), and healthy control (HC; n=12) with no atopic history (Table S2).

**RNA-seq analysis**

Total RNAs were isolated using the mirVana^TM^ miRNA ISOIation Kit (Ambion-1561) following the manufacturer’s protocol. The amount and integrity number (RIN) of total RNA were evaluated using the Agilent 2100 Bioanalyser (Agilent Technologies, Santa Clara, CA). The samples with RIN ≥ 7 were subjected to the subsequent analysis. The RNA-seq library was prepared using Illumina HiSeq^TM^ 2500 at the Shanghai Ouyi Biomedical Technology Co., Ltd. Each sample was sequenced to generate paired-end 125 bp/150 bp reads.

**Cytokine, cell signature, and functional inference analysis**

To investigate cell type abundances in PN, xCell analysis (https://xcell.ucsf.edu/#) was performed on RNA-seq data of lesional and perilesional PN skin. We reanalyzed RNA-seq data of HC normal skin and lesional PN, AD, and psoriasis skin to determine the major itch-related genes involved in PN lesion. Raw data (raw reads) were processed using Trimmomatic for quality control. Read counts were normalized based on FPKM. *P*<0.05 and fold change>2 or fold change<0.5 were set as the threshold for significantly differential expression.

**Immunofluorescence staining**

Immunofluorescence (IF) staining was performed on 3-mm-thick paraffin-embedded skin tissue sections. The sections were deparaffinized and subjected to heat-mediated antigen retrieval in 10 mmol/L citrate buffer (pH = 6.0) or EDTA buffer (pH = 9.0) in a microwave oven. The sections were blocked with normal rabbit serum in phosphate buffered saline (ZSGB-BIO, ZLI-9025) or 3% bovine serum albumin, incubated with primary antibody mix (mouse monoclonal anti-CD1a antibody at 1:400, Immunoway, YM6148 and rabbit monoclonal anti-CD207 antibody at 1:200, Abcam, ab192027; or mouse monoclonal anti-CD11c antibody at 1:1500, Proteintech, 60258-1-Ig and rabbit monoclonal anti-CD1c antibody at 1:500, Abcam, ab246520; or mouse monoclonal anti-CD11c antibody at 1:1500, Proteintech, 60258-1-Ig and rabbit polyclonal IL-31 antibody at 1:600, Immunoway, YT5841) at 37°C for 1 h, washed, and incubated with the appropriate secondary antibody mix for 1 h at room temperature. The sections were also incubated with rabbit polyclonal IL-31RA antibody (Abcam, ab113498) and rabbit polyclonal OSMR antibody (Proteintech, 10982-1-AP) at a dilution of 1:500 and 1:20, respectively, and then with the appropriate secondary antibody mix for 1 h. Nuclei were stained with DAPI (Solarbio, C0065).

**Cytokine assay**

According to the manufacturer’s protocol, we used LEGENDplex^TM^ Multi-Analyte Flow Assay Kit (BioLegend, Cat. No. 75475) to assess the levels of 12 human cytokines, including IL-2, IL-4, IL-5, IL-6, IL-9, IL-10, IL-13, IL-17A, IL-17F, IL-22, IFN-γ, and TNF-α. Furthermore, following the manufacturer’s instructions, we quantified protein expression levels of IL-31 in patients with PN and AD using enzyme-linked immunosorbent assay kits (BioLegend, Cat. No. 445707) in the serum of HC and patients with PN and AD.

**Fluorescence *in situ* hybridization combined with immunofluorescence staining**

The paraffin-embedded sections were fixed and dehydrated. Proteinase K (Thermo Fisher, AM2548) was added to digest tissues at 37°C for 2 min. The sections were incubated with the pre-hybridization solution at 37°C for 1 h. RNA *in situ* hybridization was performed using the *in situ* hybridization instrument overnight and then washed. The probe for IL-31 (5'-CCAGGCAGCAGAACAGAAAGAGCACAGACG-3') was pre-designed and purchased directly from the manufacturer (Sangon Biotech). The tissue sections were incubated with normal rabbit serum in phosphate buffered saline to block non-specific binding. After *in situ* hybridization signal detection, the sections were incubated with CD11c (1:750), CD3 (1:100), and CD68 (1:200) primary antibodies at 4 °C overnight. The sections were then incubated with the appropriate secondary antibody for 50 min. DAPI staining was performed as described earlier. These slides were scanned at ×400 magnification on a Panoramic 250 scanner (3DHistech). Each region of interest was captured was observed in five images using Panasonic software. For IF images, the Image J software was used for semi-quantitative analysis.

**Statistical analyses**

Statistical analyses were performed using GraphPad Prism (version 8.0) (GraphPad, San Diego, CA). *p*-values were determined for paired comparisons using Wilcoxon tests or unpaired comparisons using Kruskal-Wallis tests. *p* <0.05 was deemed to indicate statistical significance. GraphPad Prism was used to prepare graphs.

**REFERENCES**

1. Hanifin JM. Diagnostic criteria for atopic dermatitis: consider the context. *Arch Dermatol*. Dec 1999;135(12):1551.

2. Roh YS, Marani M, Choi U, et al. Validation of International Classification of Diseases Tenth Revision code for prurigo nodularis. *J Am Acad Dermatol*. Oct 22 2021;doi:10.1016/j.jaad.2021.10.026

3. Reich A, Chatzigeorkidis E, Zeidler C, et al. Tailoring the Cut-off Values of the Visual Analogue Scale and Numeric Rating Scale in Itch Assessment. *Acta Derm Venereol*. Jun 9 2017;97(6):759-760. doi:10.2340/00015555-2642

**
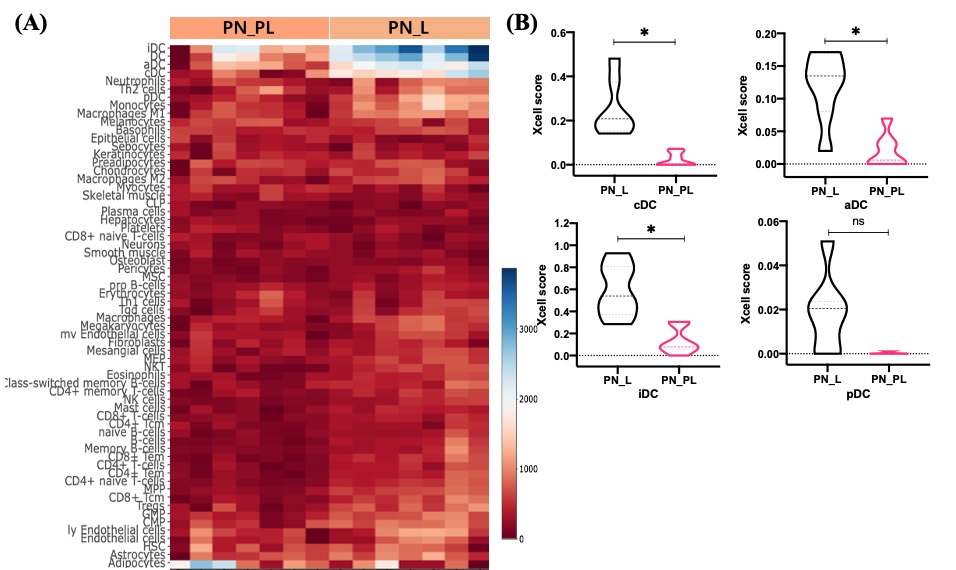
Figure S1** Prediction of dendritic cell enrichment using transcriptomic data of lesional prurigo nodularis (PN) skin with the xCell online tool. (A) Differentially infiltrated immune cells in lesional PN (PN_L) skin and perilesional PN (PN_PL) skin in patients with PN were analyzed by xCell (n=7). (B) xCell analysis demonstrated changes in dendritic cell gene signatures in PN_L and PN_PL. aDCs, activated dendritic cells; cDCs, xonventional dendritic cells; iDCs, immature dendritic cells; pDCs, plasmacytoid dendritic cells. **p*<0.05 by Wilcoxon matched-paired test; ns, not significant.


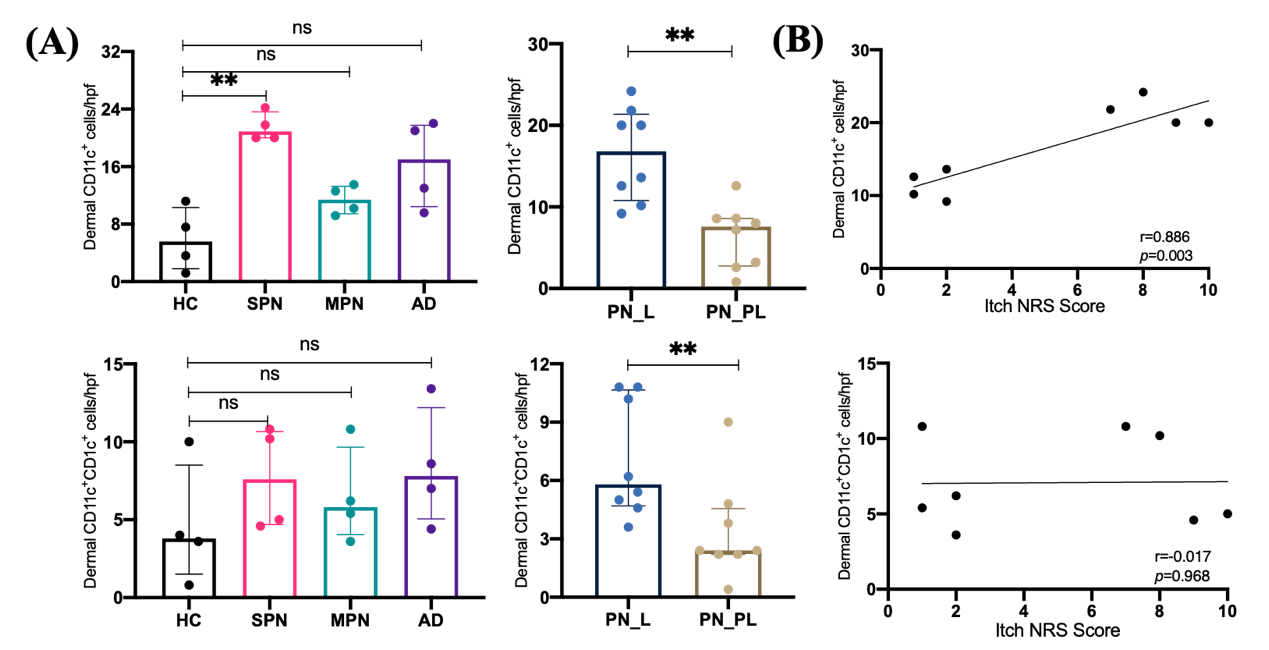


**Figure S2** The numbers of CD11c^+^ cells in the dermis in lesional prurigo nodularis (PN) skin increased and closely correlated with itch intensity. (A) Quantitation of CD11c^+^ mDCs and CD11c^+^CD1c^+^ resident myeloid dendritic cells (mDCs) in each high power field (HPF) image of the dermis (n=4, per group). n=4, ***p*<0.001 by Kruskal‒Wallis test; n=8, ***p*<0.001 by Wilcoxon matched-paired test; ns, not significant. (B) Correlation between itch Numeric Rating Scale (NRS) score and the numbers of mDCs and resident mDCs. The number of cells was counted and averaged across five randomly selected HPF images per skin sample. All data are expressed as the median with interquartile range. HC, healthy control; PN, prurigo nodularis; SPN, PN with severe pruritus (itch NRS score≥7 points); MPN, PN with mild pruritus (itch NRS score<3 points); L, lesional; PL, perilesional; AD, atopic dermatitis.


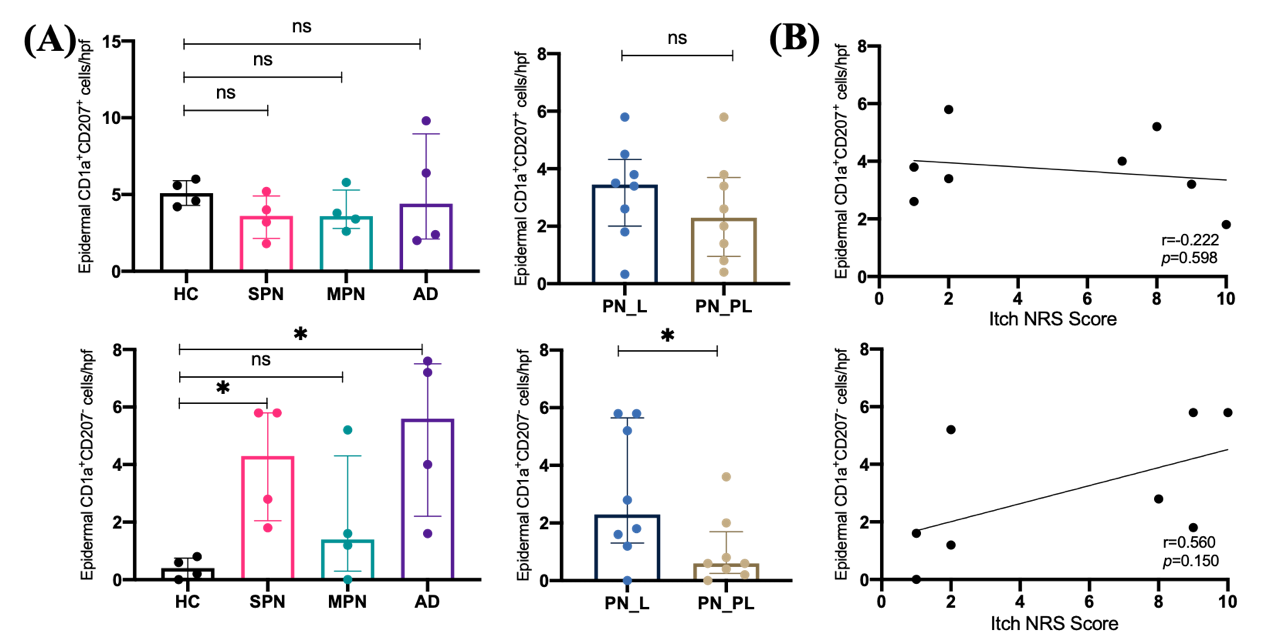


**Figure S3** The number of inflammatory dendritic epidermal cells (IDECs) increased in the epidermis in lesional prurigo nodularis (PN) skin but did not correlate with the severity of pruritus. (A) Quantification of CD1a^+^CD207^+^ LCs and CD1a^+^CD207^-^ IDECs in the epidermis per high power field (HPF) image (n=4, per group). n=4, **p*<0.05 by Kruskal‒Wallis test; n=8, **p*<0.005 by Wilcoxon matched-paired test; ns, not significant. (B) Correlations between the number of Langerhans cells (LCs) and IDECs and itch Numeric Rating Scale (NRS) score. The number of cells was counted and averaged across five randomly selected HPF images per skin sample. All data are expressed as the median with interquartile range. HC, healthy control; PN, prurigo nodularis; SPN, PN with severe pruritus (itch NRS score≥7 points); MPN, PN with mild pruritus (itch NRS score<3 points); L, lesional; PL, perilesional; AD, atopic dermatitis.


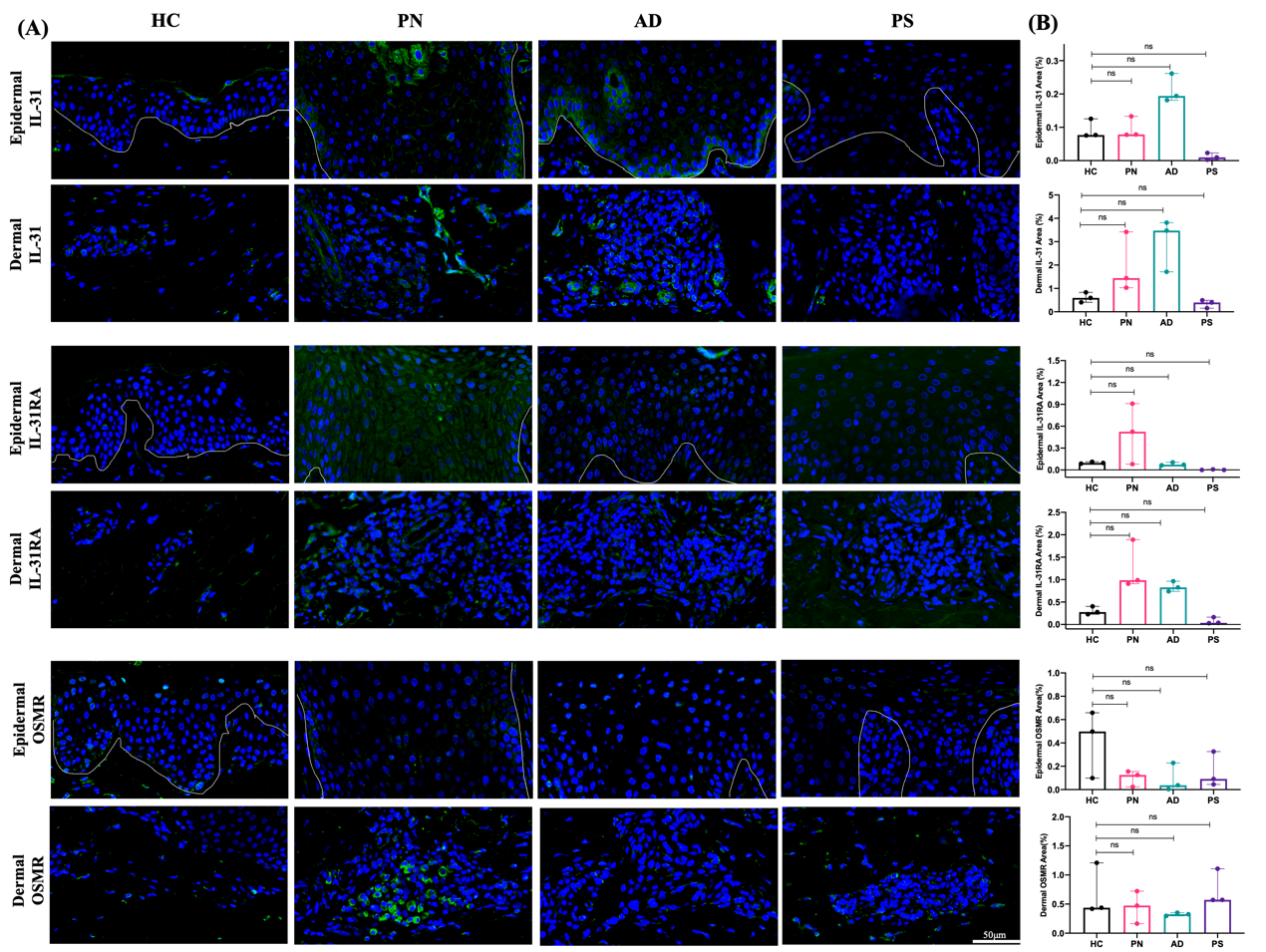


**Figure S4** The IL-31 expression in patients with prurigo nodularis (PN), atopic dermatitis (AD) and psoriasis (PS), and healthy control (HC) skin samples. (A) Representative images of IL-31 (green), IL-31RA (green), and OSMR (green) immunofluorescence staining on paraffin-embedded tissues of HC (n=3), PN (n=3), AD (n=3), and PS (n=3) samples. Nuclei were stained with DAPI (blue). Dotted lines mark the basement membrane separating the epidermis from the dermis. (B) Relative quantitation of protein levels of IL-31, IL-31RA, and OSMR in HC, lesional PN, AD, and PS skin. The levels were analyzed by Image J and averaged across five randomly selected high power field images per skin sample. All data are expressed as the median with interquartile range. ns, not significant.


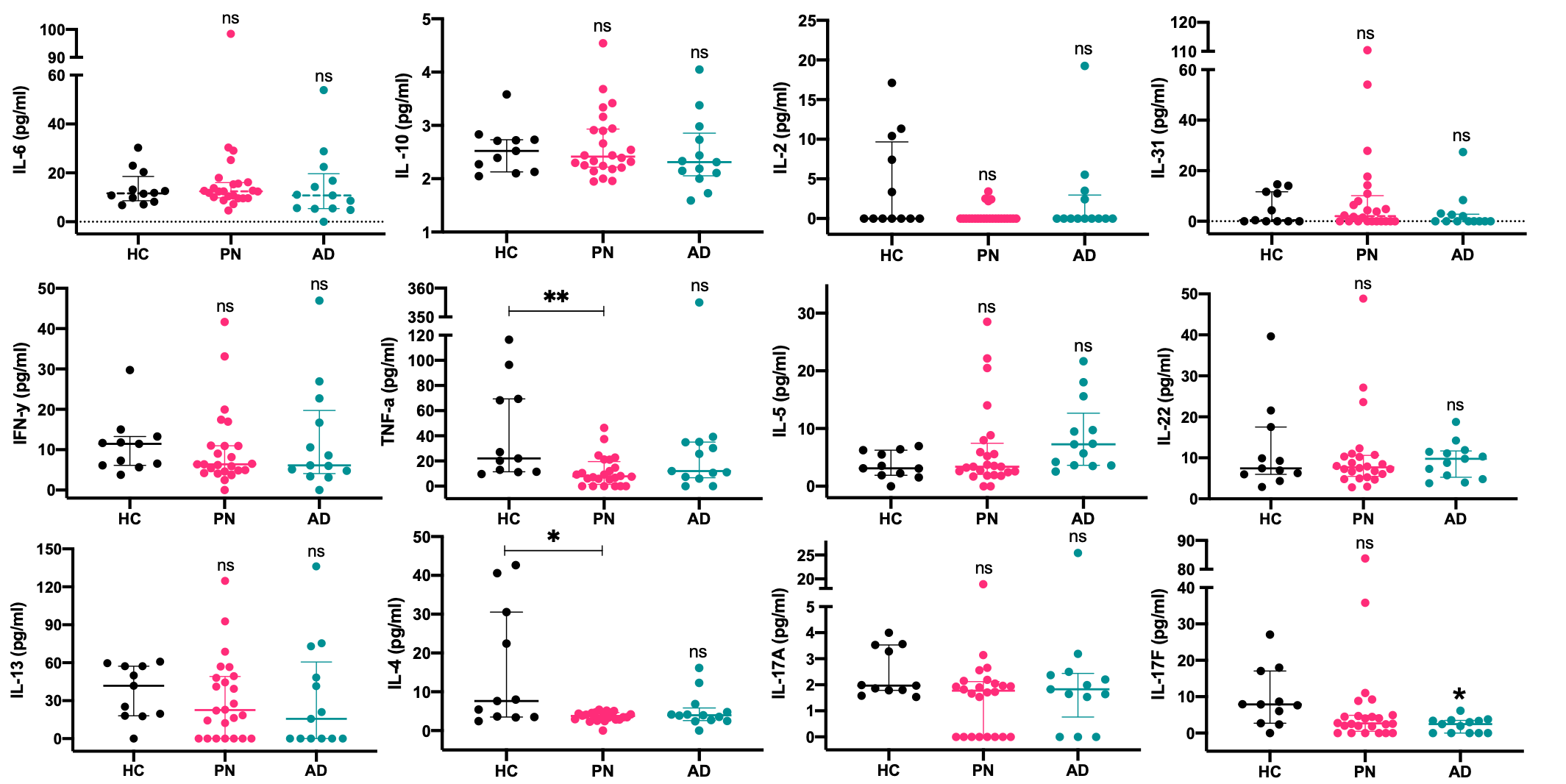


**Figure S5** Serum levels of proinflammatory cytokines in patients with prurigo nodularis (PN) and atopic dermatitis (AD) and healthy control (HC) serum samples. Proinflammatory cytokines in the serum from patients with PN (n=24) and AD (n=12) and HC (n=13) were detected using Th cytokine panel and enzyme-linked immunosorbent assay. All data are expressed as the median with interquartile range. **p*<0.05 and ***p*<0.001 by Kruskal‒Wallis test; ns, not significant.

**Table S1**. Characteristics of Patients and Healthy Control to Provide Skin Samples for mRNA, Immunofluorescence, and Fluorescence *in situ* Hybridization Combined with Immunofluorescence Staining Analyses.

| No. | Age (y) | Gender | Biopsy Site | Itch NRS Score | No. | Age (y) | Gender | Biopsy Site | Itch NRS Score |
| --- | --- | --- | --- | --- | --- | --- | --- | --- | --- |
| Patient with PN 1 | 23 | F | Leg | 1 | Patient with PS 1 | 46 | M | Leg | 5 |
| Patient with PN 2 | 49 | M | Leg | 2 | Patient with PS 2 | 61 | M | Back | 3 |
| Patient with PN 3 | 20 | F | Leg | 1 | Patient with PS 3 | 34 | M | Leg | 7 |
| Patient with PN 4 | 47 | M | Arm | 2 | Patient with PS 4 | 42 | M | Back | 4 |
| Patient with PN 5 | 47 | M | Arm | 10 | Patient with PS 5 | 25 | F | Back | 3 |
| Patient with PN 6 | 55 | M | Back | 7 | HC 1 | 35 | F | Back | 0 |
| Patient with PN 7 | 32 | F | Leg | 8 | HC 2 | 25 | F | Leg | 0 |
| Patient with PN 8 | 41 | M | Leg | 8 | HC 3 | 40 | M | Back | 0 |
| Patient with AD 1 | 19 | F | Back | 8 | HC 4 | 42 | M | Leg | 0 |
| Patient with AD 2 | 43 | F | Arm | 7 | HC 5 | 56 | M | Back | 0 |
| Patient with AD 3 | 31 | F | Leg | 8 | HC 6 | 65 | M | Back | 0 |
| Patient with AD 4 | 64 | M | Neck | 6 |  |  |  |  |  |
| Patient with AD 5 | 24 | M | Back | 8 |  |  |  |  |  |

PN, prurigo nodularis; AD, atopic dermatitis; PS, psoriasis; HC, healthy control; NRS, Numeric Rating Scale

Table S2. Characteristics of Patients and Healthy Control to Provide Serum Samples for Cytokine Assay Analyses.

| No. | Age (y) | Gender | Itch NRS Score | No. | Age (y) | Gender | Itch NRS Score |
| --- | --- | --- | --- | --- | --- | --- | --- |
| Patient with PN 1 | 23 | F | 1 | Patient with AD 1 | 19 | F | 8 |
| Patient with PN 2 | 49 | M | 2 | Patient with AD 2 | 43 | F | 7 |
| Patient with PN 3 | 20 | F | 1 | Patient with AD 3 | 31 | F | 8 |
| Patient with PN 4 | 47 | M | 2 | Patient with AD 4 | 64 | M | 6 |
| Patient with PN 5 | 47 | M | 10 | Patient with AD 5 | 24 | M | 8 |
| Patient with PN 6 | 55 | M | 7 | Patient with AD 6 | 31 | M | 10 |
| Patient with PN 7 | 32 | F | 8 | Patient with AD 7 | 66 | M | 8 |
| Patient with PN 8 | 41 | M | 8 | Patient with AD 8 | 18 | M | 7 |
| Patient with PN 9 | 37 | M | 5 | Patient with AD 9 | 35 | F | 10 |
| Patient with PN 10 | 50 | M | 5 | Patient with AD 10 | 25 | F | 8 |
| Patient with PN 11 | 54 | M | 2 | Patient with AD 11 | 50 | M | 6 |
| Patient with PN 12 | 48 | F | 8 | Patient with AD 12 | 41 | M | 8 |
| Patient with PN 13 | 62 | M | 8 | Patient with AD 13 | 24 | M | 10 |
| Patient with PN 14 | 81 | F | 4 | HC 1 | 35 | F | 0 |
| Patient with PN 15 | 76 | M | 8 | HC 2 | 25 | F | 0 |
| Patient with PN 16 | 31 | M | 8 | HC 3 | 40 | M | 0 |
| Patient with PN 17 | 24 | M | 4 | HC 4 | 42 | M | 0 |
| Patient with PN 18 | 53 | F | 8 | HC 5 | 56 | M | 0 |
| Patient with PN 19 | 69 | M | 10 | HC 6 | 65 | M | 0 |
| Patient with PN 20 | 40 | F | 5 | HC7 | 31 | F | 0 |
| Patient with PN 21 | 43 | F | 5 | HC8 | 47 | F | 0 |
| Patient with PN 22 | 22 | F | 8 | HC9 | 25 | M | 0 |
| Patient with PN 23 | 52 | M | 7 | HC10 | 23 | M | 0 |
| Patient with PN 24 | 71 | M | 9 | HC11 | 27 | M | 0 |
|  |  |  |  | HC12 | 25 | F | 0 |

PN, prurigo nodularis; AD, atopic dermatitis; HC, healthy control, NRS, Numeric Rating Scale.
